# Study on Vertical Non-Uniformity of Plasma Electrolytic Polishing

**DOI:** 10.3390/ma19132849

**Published:** 2026-07-03

**Authors:** Ziyuan Zhu, Hongtao Li, Xuchen Lu, Chao Zhang

**Affiliations:** College of Materials Science and Engineering, Nanjing Tech University, Nanjing 211816, China; zhuziyuan0815@163.com (Z.Z.);

**Keywords:** plasma electrolytic polishing, vapor gaseous envelope, non-uniformity, cavitation, oxidation

## Abstract

Aiming at non-uniformity in the vertical direction in the polishing effect on stainless steel after plasma electrolytic polishing (PEP), this paper took 304 L stainless steel as the research object. Under an ammonium sulfate electrolyte system with a mass fraction of 2.5 wt%, PEP was carried out utilizing different placement methods for the anode and electrolyte temperatures, and the causes of non-uniformity in the polishing process were explored. Experimental results demonstrate that the vertical polishing inhomogeneity originates from the upward movement of unruptured bubbles at the sample bottom. Under the combined effects of electrolyte internal pressure and bubble buoyancy, a vapor-gas envelope (VGE) featuring a thick upper part and thin lower part forms near the sample surface. This enhances plasma-related physicochemical reactions at the sample bottom and consequently raises the polishing rate. The vertical polishing unevenness can be alleviated by adjusting the electrolyte temperature. Non-uniformity could be improved by controlling the temperature of the electrolyte. Compared with the result at 95 °C, the maximum dimensional variation in each region on the sample at 75 °C was reduced by 36% because a VGE with more uniform thickness was formed, and a properly oxidized sparse layer helped protect the substrate from ablation and over-polishing. In addition, the removal rate of elements on the surface of stainless steel is affected by its activity due to the oxidation reaction. The high removal amount in the bottom region caused a trend of increasing Cr and decreasing Fe content percentages from the top to the bottom on the stainless-steel surface. However, the oxidation removal rate of elements is extremely fast due to the high temperature of the ionization center and strong electric field; therefore, the content percentage of each element on the surface is little changed after polishing.

## 1. Introduction

Extremely complex post-processing is needed when using stainless-steel parts on account of their particular requirements for surface finishing, mechanical properties, and esthetics [1,2]. There are many disadvantages regarding traditional polishing methods, and, as such, improvements are still needed. For example, mechanical polishing or chemical–mechanical polishing are difficult processes, as is polishing complex, special-shaped structural parts (such as small holes, flow channels, bosses, etc.) due to stainless steel’s own characteristics, as well as the dust generated in the process of mechanical polishing, which will do great harm to the operators [3,4,5,6]. UV laser polishing will produce some microcracks on the machined surface, which will affect the service life of the workpiece [7,8]. Furthermore, abrasive flow polishing is inaccessible for more complex parts, such as the inner walls of long pipes or structures with many inner holes, and it is easy to cause a bell mouth at both ends of the workpiece [1,2,3,9,10,11]. Therefore, the field of modern surface polishing has turned to an electrochemical system. The electrolyte in common electrolytic polishing is mostly composed of highly volatile and corrosive acidic solutions, which have caused significant environmental pollution; it is difficult to remove elements with different potentials at the same rate, which greatly limits its application [12,13,14,15]. Considering the problems of these traditional polishing methods, environmentally friendly plasma electrolytic polishing (PEP) can process complex parts, and stands out.

Plasma electrolytic polishing (PEP) integrates oxygen plasma into a conventional electropolishing system. The metallic surface is refined through the synergistic action of multiple physical and electrochemical mechanisms: anodic dissolution, oxygen-driven oxidation, plasma discharge-induced ion bombardment, dynamic evolution of the vapor–gas envelope (VGE), and cavitation forces generated by the collapse of oxygen bubbles [16,17]. Klaus et al. [18] systematically evaluated the impact of various complexing agents on polishing performance and directly compared PEP with conventional electropolishing, confirming PEP’s advantages—including reduced environmental impact (e.g., non-toxic, low-concentration electrolytes) and significantly enhanced material removal rate and surface uniformity. Concurrently, numerous international and domestic studies [19,20,21] have optimized key PEP process parameters for stainless steel—such as treatment duration, electrolyte composition (e.g., concentration and type of phosphate or glycerol-based additives), and electrical parameters (voltage, current density, pulse mode)—to achieve superior surface integrity and nanoscale roughness control. Yang et al. [22] demonstrated that PEP effectively eliminates machining marks and micro-burrs on rifled surfaces, yielding smooth edge transitions and a highly uniform metallic luster; quantitative profilometry revealed that the peak-to-valley (PtV) height of surface profiles decreased from an initial range of −16 μm to +14 μm (i.e., 30 μm total deviation) to within ±1 μm after PEP treatment—indicating over 96% reduction in surface topographic amplitude. Cornelissen [23] investigated inner-surface PEP of stainless-steel tubes under varying applied voltages and found that voltages between 260 V and 320 V consistently yielded optimal surface quality—characterized by lower Ra values and absence of localized pitting—relative to untreated controls. In industrial practice, PEP has been successfully implemented for precision finishing of 304 stainless-steel kitchenware, medical surgical instruments, high-precision dies, and nuclear-grade stainless-steel tubing. Notably, most laboratory-scale investigations focus on austenitic stainless steels—primarily 304 and 316L—while industrial-scale applications commonly employ vertically suspended workpieces clamped at one end. However, existing domestic parameter optimization studies predominantly rely on horizontally immersed specimens and single-factor or orthogonal experimental designs; consequently, systematic empirical data—especially regarding voltage distribution, bubble dynamics, and current density gradients along the vertical axis—remain scarce. As a result, the origin and extent of vertical inhomogeneity (e.g., axial variations in roughness, gloss, or oxide layer thickness) observed after PEP treatment have yet to be mechanistically explained or quantitatively correlated with process configuration.

This paper investigates the origins of vertical surface non-uniformity arising from plasma electrolytic polishing (PEP) and seeks to establish a mechanistic understanding of the process. Specifically, we examine how workpiece orientation (horizontal vs. vertical suspension), current density, and electrolyte temperature jointly influence the formation, spatial morphology, and thickness distribution of the vapor–gas envelope (VGE). These VGE characteristics are then quantitatively correlated with resulting surface topography (via 3D profilometry) and near-surface elemental composition (via EDS mapping), thereby clarifying the VGE’s function as a dynamic, spatially heterogeneous interface that governs localized electrochemical activity, plasma stability, and mass transport during PEP.

## 2. Materials and Methods

### 2.1. Experimental Materials

PEP was performed using a 304L stainless-steel block (25.000 mm × 17.800 mm × 1.860 mm) as the base material, with the following nominal composition (wt%): C ≤ 0.03, Si ≤ 1.0, Mn ≤ 2.0, Cr 18.0–20.0, Ni 9.0–12.0, S ≤ 0.03, P ≤ 0.035, and balance Fe. The initial surface roughness of the sample was 2.300 ± 0.050 μm. Prior to PEP treatment, a 3 mm diameter through-hole was drilled at the top center of the stainless-steel specimen, using a bench drill to facilitate secure fixation onto the anode and ensure electrical conductivity. Subsequently, the specimen was ultrasonically cleaned in analytical-grade ethanol for 15 min. The surface roughness and thickness of each specimen were remeasured immediately before the experiment to verify dimensional accuracy and consistency.

### 2.2. Placement Method

**Figure 1:**
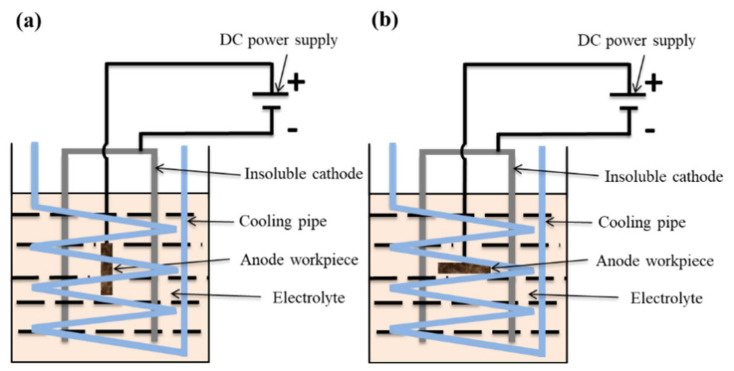
PEP device and sample placement of anode diagram: (**a**)—vertical placement; (**b**)—horizontal placement.

The experimental setup is illustrated in Figure 1. The prepared sample to be polished served as the anode in the plasma electrochemical polishing (PEP) system and was connected to the positive terminal of the DC power supply. The inter-electrode distance between the anode and cathode significantly influences the electric field strength across different regions of the sample [24], thereby causing spatial variations in the local ionization rate and ultimately affecting the polishing uniformity and efficiency. To ensure a well-defined and uniform electric field distribution, two stainless-steel plates—positioned parallel to the anodic sample—were employed as the cathode. Additionally, a cooling coil was mounted around the periphery of the stainless-steel cathode plates to regulate the temperature during polishing by controlling the flow rate of the circulating cooling water. In the configuration shown in Figure 1a, the short edge of the sample was oriented perpendicular to the electrolyte liquid surface, while the surface to be polished remained parallel to the cathode plates—hereinafter referred to as *vertical placement*. In contrast, in the configuration shown in Figure 1b, the short edge of the sample was oriented perpendicular to the parallel cathode plates, and the polishing surface was aligned parallel to the electrolyte surface—hereinafter referred to as *horizontal placement*. PEP of 304L stainless steel was conducted under both placement modes to systematically investigate the critical role of the vertical gradient of the electric field (VGE) in material removal and surface finishing. To ensure experimental consistency and comparability, when the sample was placed horizontally, it was positioned such that its central region occupied the same spatial coordinates as the central region of the vertically placed specimen. Only results obtained from this matched central location were used for direct comparison. Subsequent experiments investigating the influence of varying temperature fields were performed exclusively using the vertical placement configuration depicted in Figure 1a.

### 2.3. Experimental Parameters

The electrolyte employed in this experiment was an aqueous solution containing 2.5 wt% ammonium sulfate. Electrochemical polishing was conducted using a two-stage voltage ramping protocol: the first stage applied a constant voltage of 50 V for 30 s, followed by the second stage at 240 V for 570 s. During experiments investigating different electrode placement configurations, the electrolyte temperature was maintained at 95 °C. Unless otherwise specified, all reported temperatures refer to the peripheral (bulk) electrolyte temperature—not the localized temperature near the ionization center. In the temperature-field study, four distinct electrolyte temperatures were examined: 95 °C, 85 °C, 75 °C, and 65 °C; the total polishing duration was fixed at 10 min for all conditions. Due to the elevated operating temperature, solvent evaporation was significantly accelerated. To mitigate this, the beaker opening was sealed with a breathable preservation film featuring evenly distributed ventilation holes. This design served two purposes: (i) reducing solvent loss while (ii) facilitating gas release to suppress bubble-induced vibration and prevent accidental contact between the anode and cathode—thereby avoiding short-circuit events. Concurrently, deionized water was replenished continuously at a controlled rate of 8 mL/min throughout polishing to maintain stable electrolyte concentration and ensure experimental reproducibility and accuracy [25].

### 2.4. Characterization Method

The surface of the stainless-steel specimen was divided into top, middle, and bottom regions along the vertical direction when mounted vertically. Dimensional variations in each region before and after polishing were measured using an electronic digital display micrometer (resolution: 0.001 mm; model Nscing ES, Nscing Instrument Co., Ltd., Beijing, China). Surface roughness was quantified using a portable surface roughness tester (TR200, Xiwaka Precision Instruments Co., Ltd., Dongguan, China) with a measurement accuracy of ±0.001 μm. To ensure data reliability, ten independent roughness measurements were acquired per micro-area, and the arithmetic mean was reported as the representative value. Surface morphology before and after polishing was examined via scanning electron microscopy (SEM; JSM-IT500A, JEOL Ltd., Tokyo, Japan) to evaluate polishing efficacy and overall surface quality across the three designated micro-areas. Chemical composition changes on the stainless-steel surface were analyzed using energy-dispersive X-ray spectroscopy (EDS; detector model EX-74600U4L2Q, JEOL Ltd., Tokyo, Japan), integrated with the SEM system. Three-dimensional (3D) surface topography was characterized using a Zegage Pro white-light interferometer (Zygo Corporation, Middlefield, CT, USA). Key technical specifications include a vertical resolution of 0.1 nm, lateral resolution of 0.5 μm, maximum scan area of 10 mm × 10 mm, and Z-axis measurement range of 150 μm. All measurements were conducted in vertical scanning interferometry (VSI) mode along the central axis of each specimen, with a fixed scan area of 200 μm × 200 μm and a scanning speed of 10 μm/s. Background noise was suppressed via Gaussian filtering. The acquired 3D topographic data were processed and analyzed using MetroPro software (version 10.8, Zygo Corporation, Middlefield, CT, USA).

## 3. Results

**Figure 2:**
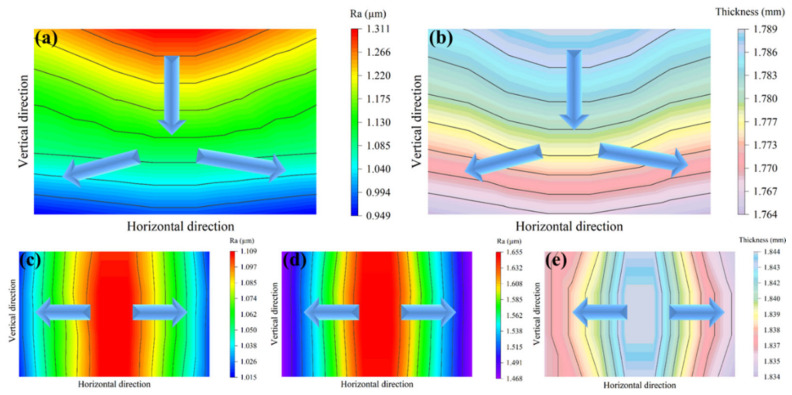
The surface roughness (**a**) and remaining thickness (**b**) of the entire specimen after polishing in vertical placement and the upper surface roughness (**c**), lower surface roughness (**d**) and remaining thickness (**e**) after polishing in horizontal placement.

Figure 2a,b presents a schematic illustration of the surface roughness and residual thickness distribution across the entire sample after micro-arc polishing of 304L stainless steel oriented vertically at 95 °C. A distinct gradient in both roughness and thickness is observed along the vertical direction—from top to bottom—and radially—from center to edges—characterized by progressively increasing surface roughness and dimensional variation. Figure 2c–e displays the corresponding surface roughness and residual thickness profiles measured on the upper and lower surfaces when the same stainless-steel sample was polished horizontally under identical conditions. Horizontal orientation yielded superior polishing uniformity compared to the vertical configuration. Specifically, the surface roughness at identical spatial coordinates was reduced to 1.109 μm on the upper surface and to 1.655 μm on the lower surface. Even at the minimum electrode spacing—where polishing efficiency is expected to be highest—the lowest achievable roughness remained as high as 1.468 μm. Nevertheless, the overall dimensional variation (i.e., thickness non-uniformity) decreased by 74.69% relative to the 83 μm observed under vertical placement.

**Figure 3:**
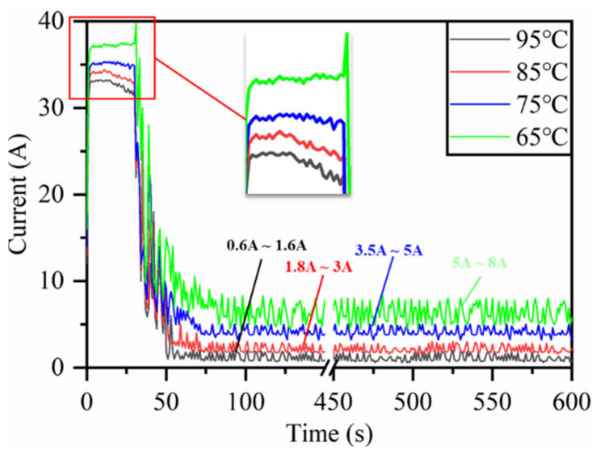
Curve of current variation during low-high stage polishing (50 V—30 s, 240 V—570 s) under various temperature fields.

The current variation curve during the PEP process at different temperatures is presented in Figure 3, with the sample oriented vertically. Regardless of temperature, the current–voltage curve exhibits two distinct regimes: a high-current regime at low voltage and a low-current regime at high voltage. Upon reaching the high-voltage stage, the current gradually stabilizes and remains steady until the end of polishing. Throughout the entire polishing process, the current at each stage progressively increases as temperature decreases, and the amplitude of current fluctuations likewise increases. Between 0 and 30 s, the current initially rises and then declines gradually; however, the duration of this gradual decline is extended at lower temperatures. Notably, at 65 °C, no discernible current decline is observed before 30 s; instead, the current displays abrupt, rapid surges and drops upon pressurization. Following the voltage increase at 30 s, the current rapidly decreases and subsequently stabilizes. The time required to reach the stable polishing stage is markedly temperature-dependent: only 24 s at 95 °C versus 50 s at 65 °C. During stable polishing, the current oscillates within a relatively high range of 5–8 A.

The total electrical resistance of the entire PEP system comprises several components: the internal resistance of the power supply, the resistance of the connecting wires, the bulk resistance of the workpiece, the interfacial resistance arising from the oxide layer formed on the workpiece surface during polishing, the resistance of the vapor–gas envelope (VGE), and the resistance of the electrolyte. Due to its extremely low electrical conductivity, the gas phase contributes negligibly to current conduction. During the process, the oxide layer on the workpiece surface undergoes partial dissolution induced by localized high temperature, while the remaining portion is mechanically removed via cavitation-induced spallation. Consequently, in comparison with the dominant resistance of the VGE, all other resistive contributions are comparatively negligible within this system.

**Figure 4:**
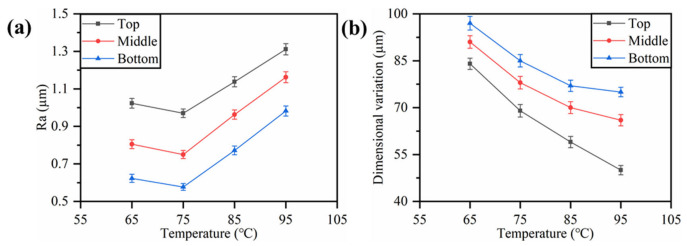
Roughness (**a**) and dimensional variation (**b**) in the middle of each micro-region after polishing under different temperature fields.

Figure 4 presents the surface roughness and dimensional variation of vertically oriented 304L stainless steel after pulsed electrochemical polishing (PEP) at different electrolyte temperatures. Here, roughness and dimensional variation refer to measurements taken at the central region of each designated area, excluding edge effects. The results indicate that polishing performance was poor in the absence of cooling water (i.e., at 95 °C), whereas it improved progressively with decreasing electrolyte temperature. At 75 °C, the average surface roughness decreased significantly—from 2.300 μm to 0.577 μm—and dimensional deviation across the three vertically aligned regions reached its minimum. Dimensional variation ranged from 69 μm to 85 μm, yielding a maximum difference of 16 μm between the top and bottom zones. In comparison, at 95 °C, the maximum regional dimensional variation was 25 μm; thus, the reduction achieved at 75 °C corresponds to a 36% decrease in discrepancy. Consequently, 75 °C was identified as the optimal polishing temperature under the present experimental conditions. Further lowering the temperature to 65 °C led to a slight deterioration in overall polishing quality, although dimensional uniformity continued to improve.

**Figure 5:**
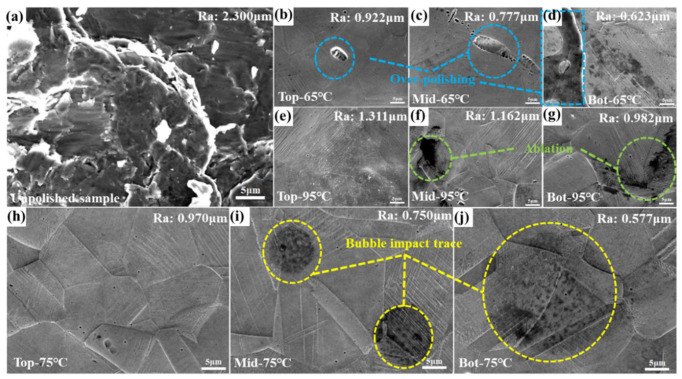
Surface morphology of the middle part of each micro-region before and after polishing under different temperature fields: (**a**)—Original sample; (**b**–**d**)—65 °C; (**h**–**j**)—75 °C; (**e**–**g**)—95 °C.

Three groups of samples—annealed at 65 °C, 75 °C, and 95 °C—were characterized by scanning electron microscopy (SEM). Figure 5 presents the surface morphology of both as-received (unpolished) samples and the central region of each micro-area following polishing at the three respective temperatures. Regardless of the annealing temperature, most regions of the stainless-steel surface transitioned from the original peak-like topography to a comparatively flat profile after polishing; notably, the polishing efficacy was superior in the bottom regions. At 65 °C, the sample surface exhibited over-polishing, manifested as pitting pits and crater-like defects (Figure 5b–d); at 95 °C, localized ablation occurred, resulting in irregular molten craters (Figure 5f,g); and at 75 °C, bubble-impact traces were observed (Figure 5i,j). Importantly, whether over-polishing, localized ablation, or bubble-impact features dominated, the top-down polishing effect remained consistently pronounced.

Subsequently, surface elemental analyses were conducted at the central location of each of the three micro-regions on the samples—both before and after polishing—at 75 °C, and the results are presented in Table 1. As shown in the table, no new elements were introduced onto the workpiece surface following polishing. Moreover, the relative content of all elements other than Cr and Fe exhibited only minor variations across all micro-regions before and after polishing, with fluctuations confined within ±0.10%. This observation indicates that plasma electrolytic polishing (PEP) does not involve directional corrosion or selective dissolution of specific elements induced solely by the electrolyte; rather, these phenomena arise from the synergistic interplay of multiple concurrent processes—including electrochemical reactions, oxidation, and cavitation.

**Figure 6:**
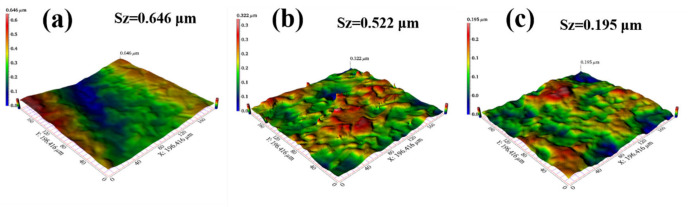
Three-dimensional profilometry images of different regions at 75 °C: (**a**) upper region, (**b**) middle region, (**c**) bottom region.

Figure 6 presents the 3D surface topography and the corresponding maximum peak-to-valley height (Sz) values measured at the upper (a), middle (b), and bottom (c) regions along the axial direction of vertically clamped 304L stainless steel after plasma electrolytic polishing (PEP) at an electrolyte temperature of 75 °C. As revealed by both the contour nephograms and quantitative Sz data, surface undulation decreases progressively from top to bottom along the vertical axis. The upper region exhibits the largest height variation, with an Sz value of 0.646 μm, and is characterized by extensive alternating protrusions and pits and pronounced macroscopic surface inhomogeneity. At the middle region, the Sz value decreases to 0.522 μm, accompanied by reductions in the number and amplitude of surface bulges and diminished profile fluctuation. In contrast, the bottom region—located adjacent to the electrolyte–air interface—exhibits a significantly lower Sz of only 0.195 μm, and features a mild, compact, and highly planarized surface morphology, thereby achieving the optimal planarization effect.

This axial morphological gradient arises from the spatially non-uniform evolution of the vapor–gas envelope (VGE) and bubble distribution during vertical PEP. Hydrogen–oxygen mixed bubbles generated by plasma discharge migrate upward under buoyancy and accumulate preferentially on the upper specimen surface, resulting in a thicker and inherently unstable VGE. Frequent local VGE breakdown and transient, spatially fluctuating discharges consequently intensify non-uniform material dissolution, yielding a severely undulating surface topography. Conversely, in the bottom region—proximal to the bulk electrolyte—bubble accumulation is markedly suppressed, enabling the formation of a uniform and stable VGE. Under these conditions, electrochemical and plasma-assisted material removal proceeds more steadily and homogeneously, leading to superior surface flatness relative to the upper and middle regions. The quantitative 3D profilometry results thus provide direct experimental evidence for the intrinsic axial inhomogeneity in polishing performance observed for vertically oriented samples processed at a fixed electrolyte temperature of 75 °C.

**Figure 7:**
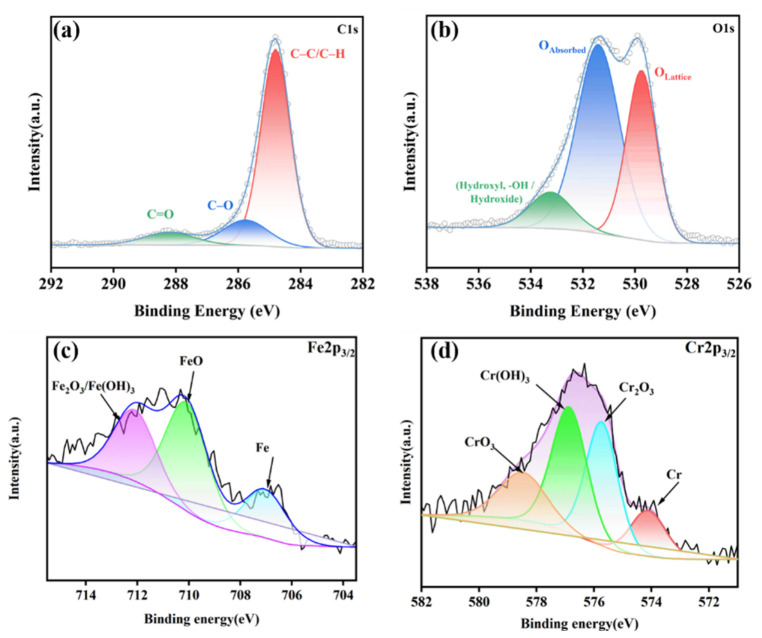
High-resolution XPS spectra of various elements after plasma electrolytic polishing: (**a**) C 1s, (**b**) O 1s, (**c**) Fe2p_3/2_, (**d**) Cr2p_3/2_.

High-resolution X-ray photoelectron spectroscopy (XPS) measurements were performed to elucidate the chemical states of elements on the PEP-modified surface; the corresponding spectra are shown in Figure 7.

For the C 1s spectrum (Figure 7a), distinct characteristic peaks assigned to C–C, C–O, and C=O bonds are observed. Given that the ammonium sulfate electrolyte employed in this study contains no organic constituents, the detected carbon signal arises exclusively from adventitious hydrocarbon contamination adsorbed from the ambient atmosphere onto the freshly polished, reactive metal surface. This accounts for the slightly elevated surface carbon content relative to that of the untreated substrate. 

The O 1s spectrum (Figure 7b) was deconvoluted into three well-resolved components based on established binding energy references. The peak centered at 529.5–531.0 eV is attributed to lattice oxygen in Fe–Cr mixed metal oxides. The component appearing at 531.0–532.2 eV corresponds to oxygen in oxide species (e.g., M–O, where M = Fe or Cr), while the higher-binding-energy component (532.2–533.5 eV) is assigned to surface hydroxyl groups (–OH). As reported in prior literature, hydroxyl groups exhibit the highest binding energy among all oxygen-containing species—a trend fully consistent with our spectral-fitting results. It should be noted that XPS cannot directly detect oxygen vacancies; instead, shifts in the O 1s peak position and changes in its relative intensity reflect variations in the chemical bonding environment of oxygen atoms within the surface oxide film—particularly differences in the nature and strength of oxygen–metal interactions.

The Fe2p_3/2_ spectrum (Figure 7c) exhibits multiple overlapping peaks. The primary features in the binding energy range of 700–714 eV are deconvoluted into contributions from metallic iron and multivalent iron oxides. Consistent with established XPS spectral interpretation guidelines, satellite peaks are explicitly included in the fitting for features appearing above 714 eV. The optimized deconvolution curve closely reproduces the experimental spectrum, confirming the coexistence of metallic Fe and iron oxides on the polished surface.

For the Cr2p_3/2_ spectrum (Figure 7d), the fitted components corroborate the formation of chromium oxides on the specimen surface. In conjunction with the morphological observations, the non-uniform intensity distribution across the Cr2p_3/2_ peaks further supports the presence of a vertically heterogeneous oxide film thickness. The dense, predominantly Cr_2_O_3_-based chromium oxide layer functions as a stable passive film and governs the interfacial dissolution behavior during plasma electrolytic polishing (PEP).

## 4. Discussion

### 4.1. Mechanism of PEP

**Figure 8:**
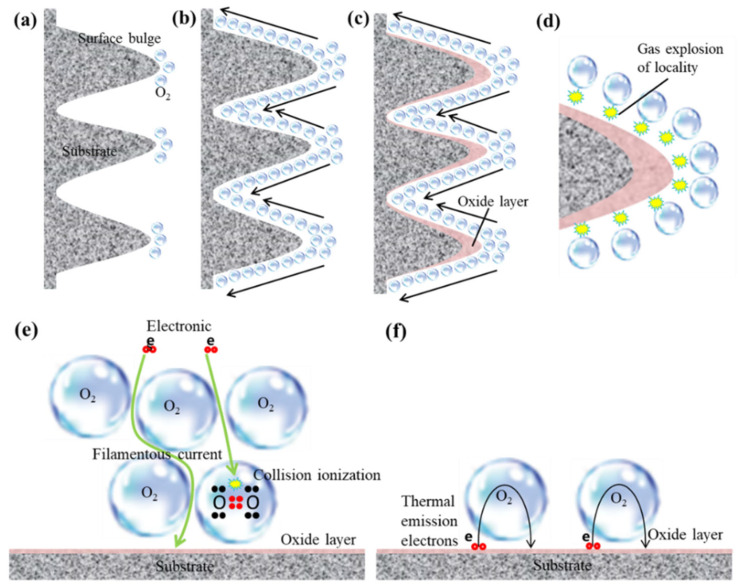
Model of PEP process: (**a**)—point effect; (**b**)—extended surface discharge; (**c**)—oxidized-sparse layer; (**d**)—gas explosion of locality; (**e**)—filamentary current and collision ionization; (**f**)—thermal electron emission.

At the moment of electrification, water electrolysis initiates preferentially at surface protrusions due to the point effect, generating oxygen and thereby inducing localized resistance imbalance within the microregion [26,27]. Driven by curvature-dependent electrochemical kinetics, the electrolytic reaction spontaneously propagates from regions of high curvature (i.e., convex features) toward those of lower curvature (i.e., concave regions). This progressive front ultimately yields a relatively stable, fully enveloping vapor–gas envelope (VGE) over the sample surface (Figure 8b). The formation and evolution of this VGE give rise to a negative differential resistance (NDR) effect [28], resulting in characteristic current fluctuations followed by a gradual decline—manifested as an initial current rise followed by a slow decrease during the 0–30 s interval (Figure 3).

Throughout the electropolishing process, the generated oxygen continuously reacts with metallic elements on the anode surface to form metal oxides. Following the rapid voltage increase at 30 s, a substantial surge in oxygen evolution occurs. Under the insulating barrier of the VGE, electrons accumulate at the VGE–electrolyte interface and are accelerated toward the anode surface under the strong applied electric field. A fraction of these electrons traverse the sample surface via low-resistance, filamentary conduction pathways through oxygen-filled gaps (Figure 8e); the remainder undergo collisional ionization upon interacting with oxygen molecules. Under the influence of the intense electric field, oxygen molecules experience field-assisted ionization, triggering an electron avalanche and thereby enhancing plasma generation—yielding a dense oxygen plasma layer.

The fully filled valence-shell electron configuration of chromium imparts exceptional thermodynamic stability to the resultant Cr_2_O_3_ passive film. Chromium atoms preferentially occupy outer electron orbitals, facilitating the formation of a compact, continuous passive oxide layer that effectively suppresses corrosive dissolution of the underlying metallic substrate [29]. The intense thermal energy engendered by ionization imparts sufficient kinetic energy to the delocalized surface electrons—surpassing their work function threshold—thereby propelling them into a thermionic emission regime wherein they surmount the conduction band barrier and desorb from the sample’s surface (Figure 8f). These liberated electrons, subsequently accelerated by the localized electric field within their mean free path, undergo secondary ionization upon energetic collision with nascent oxygen microbubbles. Concurrently, the elevated temperature induces thermal expansion of the surrounding non-ionized oxygen, while vigorous convective currents—driven by steep thermal gradients between hot ionization zones and cooler electrolyte regions—trigger rapid bubble collapse (Figure 8d). This dynamic interplay culminates in the generation of a Bjerknes force [30], directed perpendicularly toward the oxide layer’s interface, thereby enabling precision electrochemical polishing.

Moreover, the magnetic field arising from current flow through the transient plasma exerts a centrifugal magnetic compression effect [31] upon the ionized domain, simultaneously amplifying localized thermoelastic stress within the thermally inflated bubbles—precipitating controlled micro-explosions. Under the synergistic influence of rapid bubble nucleation, progressive interfacial thickening, violent implosive collapse, and sustained electron ejection, the entire system resides in a pronounced non-equilibrium state. After 30 s, the voltage-governed electrochemical double-layer (VGE) progressively thickens with increasing applied potential, inducing current oscillations ethat gradually attenuate and ultimately converge toward a quasi-steady-state equilibrium.

### 4.2. Generation of Vertical Direction Non-Uniformity

**Figure 9:**
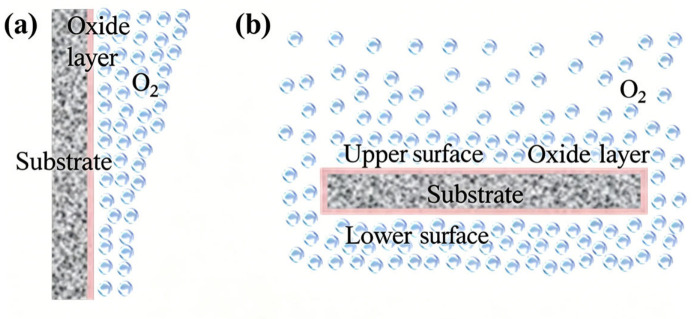
Models of VGE during polishing with different placement methods: (**a**)—vertical placement; (**b**)—horizontal placement.

The vertical non-uniformity observed when the sample is oriented upright stems from the excessive accumulation of oxygen bubbles beneath its surface during electropolishing. Under the combined influence of electrolyte-induced internal pressure and inherent buoyancy, certain intact oxygen bubbles ascend steadily—indeed, copious bubble generation and continuous effervescence are hallmarks of this electrochemical process—thereby engendering a trapezoidal, oxygen-enriched plasma-like virtual galvanic electrode (VGE) atop the sample surface; the VGE is thickened at the top and progressively attenuated toward the bottom (Figure 9a). This spatially graded VGE architecture yields a corresponding gradient in local electrical resistance: thinner regions exhibit markedly reduced resistivity, thereby channeling a concentrated electron flux that intensifies electrochemical activity—including vigorous electrolysis, pronounced oxidation, robust ionization, and even localized cavitation. Consequently, the degree of material removal—manifested as escalating surface roughness and dimensional deviation—progresses systematically from top to bottom. Yet, the geometric singularity at the sample’s periphery—characterized by a sharply diminished curvature radius—induces significant charge concentration along the edge [32], thereby amplifying electrochemical reactivity radially outward. This edge-enhanced field distribution drives a concurrent horizontal non-uniformity: polishing intensity escalates progressively from the central region toward the outer margins. In contrast, when the sample is positioned horizontally with its X-axis aligned along the long dimension, the roughness and dimensional variation across all Y-axis (short-dimension) cross-sections—corresponding to each discrete X-coordinate—remain remarkably uniform (Figure 2c–e). This homogeneity arises because non-ruptured oxygen bubbles residing on the upper surface exhibit negligible lateral migration; instead, they escape exclusively through the electrolyte–air interface, gradually depleting local bubble density. Meanwhile, bubbles adhering to the lower surface either detach and rise laterally from the sample’s edges (Figure 9b), or—under the physical constraint imposed by the sample itself—coalesce into thicker, more persistent layers. Such stagnation severely impedes mass transport and charge transfer, drastically curtailing polishing efficacy in the lower region. Ultimately, the dynamics of this dichotomous bubble yield a striking top–bottom asymmetry: superior surface finish (lower roughness) on the upper face, juxtaposed against inferior finish (higher roughness) on the lower face.

For the non-uniform distribution of surface elemental composition following polishing (Table 1), this phenomenon arises from the differential reactivity of constituent elements under oxygen plasma exposure. Specifically, iron—possessing inherently higher chemical activity—is preferentially oxidized by reactive oxygen species, leading to a pronounced depletion in its surface concentration. In the bottom region of the workpiece, the thinner native oxide layer, coupled with intensified ion bombardment and localized high-temperature conditions induced by vigorous plasma ionization, accelerates substrate consumption. Consequently, Fe is removed more aggressively in this zone, resulting in a distinct downward gradient in its surface atomic percentage—from top to bottom.

In contrast, chromium exhibits superior chemical inertness and intrinsically sluggish oxidation kinetics. Yet, under the synergistic influence of an intense electric field and thermally activated energy—both amplified by the high degree of plasma ionization—the oxidation of Cr is markedly enhanced. As a result, its removal rate, though marginally lower than that of Fe, remains remarkably substantial. Crucially, the comparatively modest etching rate of Cr engenders a dynamic surface evolution: before the Cr-rich subsurface layer is fully stripped away, continued preferential removal of other elements—including residual Fe—exposes fresh Cr-containing interfaces beneath. This regenerative exposure mechanism ultimately drives a net enrichment of Cr at the surface.

Accordingly, the pronounced Fe depletion in the bottom region further amplifies this effect, yielding a complementary upward-to-downward increase in Cr surface concentration across the polished workpiece. Nevertheless, it bears emphasis that the overall oxidation–stripping kinetics for all surface elements proceed at an exceptionally rapid pace; thus, the relative disparity between Fe and Cr removal rates—though mechanistically significant—exerts only a subtle modulating influence on the final compositional outcome.

### 4.3. Effects of Electrolyte Temperature on Non-Uniformity

The reduction in electrolyte temperature intensifies both high- and low-temperature convection currents during the electropolishing process, thereby accelerating the dynamic collapse of gas bubbles. Concurrently, the lowered thermal energy diminishes the solution’s electrical conductivity, significantly retarding ion migration kinetics and consequently suppressing the rate of the anodic electrolytic reaction—leading to a marked deceleration in oxygen bubble nucleation. Moreover, excessive thermal accumulation at the ionization core induces localized electrolyte vaporization; however, upon introduction of cooling water, the solution’s gasification rate plummets dramatically. Ultimately, under the synergistic influence of sharply curtailed generation rates for both oxygen and vapor bubbles, the vapor–gas envelope (VGE) struggles to sustain structural integrity. As a result, the VGE—once fully enveloping the stainless-steel surface—becomes markedly thinner and more tenuous. Furthermore, the time required for VGE stabilization during the extended surface-discharge phase is substantially prolonged. At 95 °C, the initial VGE forms rapidly within merely 15 s; yet at 75 °C, this formation time extends to 21 s. Strikingly, even under applied pressure at 65 °C—maintained for a full 30 s—the VGE remains incapable of achieving complete, uniform coverage over the anodically polarized stainless-steel surface. At the instant voltage is applied, this incompletely formed, inherently fragile VGE exhibits extreme metastability—promptly undergoing dielectric breakdown and triggering an abrupt, steep surge in current (Figure 3). Nevertheless, despite this instability, substantial oxygen evolution persists, continuously reconstituting the gaseous sheath around the stainless-steel surface, sustaining oxygen plasma generation, and perpetuating bubble-collapse-driven polishing. Consequently, the current still undergoes a rapid decline—yet the precarious VGE architecture engenders severe current oscillations, causing violent fluctuations between 10 and 16 A throughout the decay phase.

The diminution in bubble quantity induced by low-temperature conditions effectively narrows the inter-microregional disparity in bubble density. Consequently, the vertically asymmetric voltage gradient distribution—characterized by a thicker upper zone and a thinner lower zone—progressively evolves toward a more uniform, rectangular configuration. This morphological transition engenders a marked homogenization of the electropolishing efficacy across all microregions. As such, decreasing temperature correlates directly with enhanced dimensional uniformity among distinct surface domains.

Although reduced thermal energy inevitably decelerates both the electrolytic decomposition kinetics and the oxidative reaction rates, it concurrently amplifies cavitation-driven stripping efficiency—thereby accelerating the detachment and shedding of the nascent oxide layer. Crucially, the implosive collapse of bubbles transmits intense, localized mechanical forces directly onto the substrate surface; heightened cavitation utilization, therefore, progressively exacerbates dimensional nonuniformity in stainless steel—as evident in Figure 4b.

At an electrolyte temperature of 65 °C, bubble nucleation plummets precipitously, inducing pronounced discontinuity within the voltage gradient envelope (VGE). This instability fosters dynamic fluctuations across the surface’s microtopography: the electric field concentration at microscale protrusions—particularly at asperity tips—is significantly attenuated, thereby weakening the preferential discharge effect. Nevertheless, the overarching polishing mechanism remains governed by topographic selectivity—i.e., preferential material removal at peaks and ridges persists as the dominant paradigm.

Conversely, micro-pits accumulate comparatively higher bubble densities; these bubbles undergo vigorous oscillation and rapid egress, facilitating greater current density penetration and thus intensifying substrate dissolution in those recessed zones. The net result is a markedly increased surface roughness in the polished stainless steel. Notably, although bubble generation at this elevated temperature is sparse, the residual population remains sufficient to sustain the minimum VGE threshold requisite for effective electropolishing. Moreover, near the sample’s basal region, lingering bubbles—delayed in collapse and ascending buoyantly—induce vertical stratification in the final surface morphology, culminating in pronounced through-thickness nonuniformity post-polishing.

At an electrolyte temperature of 65 °C, the electric field exerts a markedly enhanced influence—attributable to the diminished thickness of the vapor–gas envelope (VGE)—thereby inducing a discernible over-polishing effect (Figure 5b–d). Conversely, under a thermal regime of 95 °C, the ionization core sustains persistently elevated temperatures, effectively suppressing the establishment of efficient convective heat transfer. On one hand, intensified gasification ensues, while the thermally induced surge in electrical conductivity markedly accelerates the kinetics of the electrolytic reaction. Simultaneously, the attenuated thermal gradient decelerates bubble collapse dynamics, culminating in a pronounced thickening of the VGE. Although oxidation proceeds unabated, the resultant oxide layer remains impervious to effective cavitation-induced mechanical impact—thus impeding productive material removal and diminishing surface roughness modulation. On the other hand, the ionization core’s excessive thermal energy—unable to dissipate promptly—generates substantial resistive heating within the increasingly insulating, thickened VGE. This synergistic thermal–electrochemical feedback loop drives the stainless-steel surface to extreme localized temperatures, ultimately triggering non-negligible ablation (Figure 5f,g). While the comparatively low-resistance, thinner VGE at lower regions engenders only modest Joule heating, the attendant cavitation-driven stripping of the underlying oxide layer exposes the pristine substrate—rendering it acutely vulnerable. Moreover, thermal conduction from the intensely heated top region propagates downward, further sensitizing the substrate to ablation. Although the top region exhibits heightened roughness—owing to the accumulation of a robust, passivating oxide layer—it benefits from exceptional protection against substrate erosion, thereby preserving structural integrity despite diminished polishing efficacy. At 75 °C, the localized temperature undergoes a pronounced reduction, while the ultrathin vapor-generating envelope (VGE) and the sparsely oxidized layer—residually deposited by transient bubble cavitation—coalesce into a synergistic protective barrier. Consequently, both the electric-field–driven and thermally induced degradation mechanisms are substantially attenuated; as a result, no ablation features or corrosion pits manifest across the top, middle, or bottom micro-regions following electropolishing. Nevertheless, the implosive collapse of bubbles situated at the innermost interfacial zone engenders localized hydrodynamic shockwaves, leaving discernible cavitation impact imprints on the substrate surface (Figure 5i,j). This observation unequivocally underscores the pivotal—and indeed indispensable—role played by cavitation-induced mechanical forces in stainless steel pulsed electropolishing (PEP).

## 5. Conclusions

In this study, plasma electrolytic polishing (PEP) was meticulously executed on 304L stainless-steel substrates, employing an inorganic ammonium sulfate-based electrolyte. Through deliberate optimization of the anode clamping architecture and precise thermal regulation of the electrolyte—complemented by a multifaceted suite of advanced characterizations including scanning electron microscopy (SEM), energy-dispersive X-ray spectroscopy (EDS), three-dimensional surface profilometry, and site-specific X-ray photoelectron spectroscopy (XPS)—the fundamental physicochemical origins underlying axial polishing nonuniformity in vertically oriented specimens were rigorously elucidated and systematically deconstructed. The principal findings are concisely distilled below:

The anode clamping configuration exerts a decisive influence over the spatial distribution of the vapor–gas envelope (VGE) and, consequently, governs the macroscopic homogeneity of the polishing process. In vertically suspended samples, buoyancy-driven upward migration of gaseous byproducts engenders a distinctly trapezoidal VGE morphology—characterized by pronounced thickness augmentation at the upper region and progressive attenuation toward the base. This gradient-induced thinning of the VGE near the specimen’s lower extremity markedly diminishes local electrical resistance, thereby amplifying plasma discharge intensity and accelerating preferential material ablation from bottom to top. In stark contrast, transitioning to a horizontal sample orientation effectively suppresses directional bubble coalescence, yielding a dramatic 74.69% reduction in dimensional deviation relative to the vertical configuration—and concurrently elevating polishing uniformity to an exceptional degree under otherwise identical experimental parameters.

Electrolyte temperature enhances axial homogeneity by modulating the morphology of the vapor-generating electrolyte (VGE) layer. Lowering the temperature suppresses electrolyte vaporization and bubble formation, thereby transforming the asymmetric trapezoidal VGE into a uniform rectangular film and alleviating axial polishing non-uniformity. Within the present experimental setup, 75 °C is identified as the optimal temperature, reducing the maximum dimensional difference between the upper and bottom regions by 36% relative to that observed at 95 °C. Excessively high temperature (95 °C) leads to an over-thick VGE layer and insufficient heat dissipation, resulting in localized substrate ablation; conversely, excessively low temperature (65 °C) destabilizes the VGE layer, causing irregular tip discharges and excessive material removal. At 75 °C, a thin and sparse oxide layer forms effectively, suppressing excessive substrate corrosion while preventing both ablation and over-polishing defects.

Variations in surface elemental composition are governed by differences in elemental oxidation kinetics. Energy-dispersive X-ray spectroscopy (EDS) results indicate that Fe—exhibiting higher oxidation activity—is preferentially removed via plasma-induced oxidation, whereas Cr forms dense, stable passive oxides and is consequently removed at a lower rate. As a result, Fe content gradually decreases and Cr content gradually increases from the top to the bottom of the specimens. However, elevated temperature and the intense electric field within the ionization zone collectively accelerate overall elemental removal and diminish the relative disparity in removal rates among elements, rendering the variation in elemental mass fractions before and after polishing negligible. Single-spot XPS analysis confirms the non-uniformity of the oxide film and further substantiates the VGE-governed polishing mechanism: following plasma electrolytic polishing (PEP), Fe–Cr composite oxide layers—incorporating lattice oxygen vacancies—are formed. Concurrent detection of signals from the underlying metallic substrate and multivalent metal oxides indicates spatially heterogeneous oxide thickness, consistent with the axial surface topography fluctuations quantified by three-dimensional profilometry. In the absence of organic additives in the electrolyte, the observed increase in surface carbon content is attributed to ambient hydrocarbon contaminants adsorbed onto the freshly activated metallic surface.

In summary, axial polishing inhomogeneity under vertical PEP is primarily governed by an axially non-uniform distribution of the virtual ground electrode (VGE) layer, rather than intrinsic differences in elemental reactivity. Therefore, achieving uniform VGE thickness across the entire specimen surface represents a critical strategy for improving polishing consistency and obtaining high-quality, defect-free finished surfaces.

## Figures and Tables

**Table 1:** Chemical composition of the middle of each micro-region on the surface of the sample, before and after polishing, at 75 °C.

Element	Before Polishing			After Polishing		
	Mass% (Top)	Mass% (Mid)	Mass% (Bot)	Mass% (Top)	Mass% (Mid)	Mass% (Bot)
C	1.17 ± 0.1	1.15 ± 0.1	1.19 ± 0.1	1.14 ± 0.1	1.09 ± 0.1	1.09 ± 0.1
Si	0.51 ± 0.1	0.50 ± 0.1	0.53 ± 0.1	0.51 ± 0.1	0.48 ± 0.1	0.44 ± 0.1
Cr	18.49 ± 1.2	18.50 ± 1.2	18.47 ± 1.2	18.64 ± 1.2	18.78 ± 1.2	18.99 ± 1.2
Mn	0.84 ± 0.1	0.88 ± 0.1	0.86 ± 0.1	0.86 ± 0.1	0.94 ± 0.1	0.77 ± 0.1
Fe	71.49 ± 2.1	71.49 ± 2.1	71.46 ± 2.1	71.37 ± 2.1	71.18 ± 2.1	71.12 ± 2.1
Ni	7.50 ± 0.1	7.48 ± 0.1	7.49 ± 0.1	7.48 ± 0.1	7.53 ± 0.1	7.59 ± 0.1

## Data Availability

The original contributions presented in this study are included in the article. Further inquiries can be directed to the corresponding author.

## Nomenclature

wt%	Weight percent
μm	Micron
PEP	Plasma electrolytic polishing
VGE	Vapor–gas envelope
